# Ship Fever

**Published:** 1847-07

**Authors:** 


*Ship Fever.*—Both Journals of Medicine and the newspapers, generally, are bringing frightful intelligence of the extension of ship fever in Europe, and all the Atlantic ports, north of Philadelphia, in this country. Vessels are continually arriving here with vast multitudes of miserable human beings, from famine-stricken Ireland, who were both physically and morally enfeebled before commencing a voyage which disease tracks across the ocean with an unerring certainty. Complaints are made that the ship fever is by no means confined to the emigrant vessels, but that it appears on shore, clinging to the Irish emigrant, and breaking him down even far in the country, after he has escaped from the confinement of a ship hold. This is true to a degree ; but had these thoughtless, head-strong, imprudent people one ray of discretion when they get on land, their sufferings would be less than they are. Cases of ship fever would be fewer in number, and less severe in character, were the emigrants influenced by the advice urged upon them by kind-hearted, benevolent physicians and others. Soon after leaving the vessel, however good their condition at the time, they seize with ravenous avidity upon every possible variety of edible that comes within their reach, to say nothing of drinks—and the result is a sudden engorgement of the stomach. Nature seeks relief from this plethora, in some instances by a diarrhœa ; in others, a peculiar state of the system is induced, remarkable for the turbulence of the blood, which seems to boil and foam in the vessels—and this is ship fever, with all its bad concomitants.

Bad food, and the huddling together of men, women and children in the hold of a ship for weeks, engender the disease which is brought to our shores. When those who have escaped the action of a poisoned atmosphere between decks, afterwards sicken on land, exhibiting the same degree of intensity in the symptoms, the disease is brought on, in a majority of cases, as already remarked, by the uncontrolled appetites of the victims to the malady.

A constant professional intercourse with multitudes of Irish emigrants, who arrive in the port of Boston, furnishes opportunities for witnessing ship fever in all its phases. The only efficient remedy, certainly the first source of relief, is a fresh atmospheric exposure. It is delightful to contemplate the changes effected by this simple process. Very little medication is required in the management of patients from sea. Of the kind of treatment most satisfactory with the other class, those sickening from over-eating, and other irregularities, there may be a variety of opinions. The disease terminates fatally, very quickly, at sea; but the worst forms, on being removed to cool, airy apartments on shore, are at once ameliorated, unless the low, muttering delirium exists. Under such circumstances, a recovery is exceedingly doubtful. Petæchial spots, referred to by foreign authors, have in no instance, thus far, been observed on those dying here.

Next—is the disease infectious? Does it re-produce itself in persons exposed to the emanations from those labouring under it? These are questions seriously agitated by the mass of the people. Many persons consider that it is not thus propagated, but that it is only generated in the manner represented in these observations. Fatigue, debility, a tainted atmosphere, or badly-ventilated apartments, together with direct exposure to those in the advanced stages of the fever, without doubt, may produce it. Hence, cases are perpetually occurring in public institutions, where foreign paupers are admitted, and in narrow streets, and old decaying tenements where emigrants congregate on reaching the city. The views of correspondents on this engrossing topic are solicited, since the public health should not be neglected by medical practitioners.—*Boston Med. and Surg. Journ.*

